# Mislabeling and nomenclatorial confusion of *Typhlotanais sandersi* Kudinova-Pasternak, 1985 (Crustacea: Tanaidacea) and establishment of a new genus

**DOI:** 10.7717/peerj.14272

**Published:** 2022-11-24

**Authors:** Marta Gellert, Ferran Palero, Magdalena Błażewicz

**Affiliations:** 1 Department of Invertebrate Zoology and Hydrobiology, Faculty of Biology and Environmental Protection, University of Lodz, Lodz, Poland; 2 Cavanilles Institute of Biodiversity and Evolutionary Biology, Valencia, Paterna, Spain; 3 Department of Life Sciences, The Natural History Museum, London, England

**Keywords:** Peracarida, North Atlantic, Deep-sea, Northwest Pacific

## Abstract

Re-examination of historical collections allowed us to resolve the taxonomic status of *Typhlotanais sandersi* Kudinova-Pasternak, 1985, originally described based on a single specimen from Great-Meteor Seamount. The holotype of this species was considered lost and the species redescribed based on a second specimen from the type locality by Błażewicz-Paszkowycz (2007a), who placed *Ty. sandersi* on a newly established genus *Typhlamia*. Thorough morphological analysis of *Typhlamia* and *Typhlotanais* species and recently obtained genetic data of typhlotanaids from N Atlantic and NW Pacific waters allow us to conclude that the redescription of *Ty. sandersi* by Błażewicz-Paszkowycz (2007a) was based on a wrongly labelled specimen that, rather than a type of *Ty. sandersi*, represents in fact a new species of *Typhlamia*. The morphological comparison of the type species of *Typhlotanais* (*Ty. aequiremis*) with all ‘long-bodied’ typhlotanaid taxa with rounded pereonites margins (*i.e*., *Typhlamia, Pulcherella, Torquella*), and the use of genetic evidence, support the establishment of a new genus to accommodate: *Ty. sandersi*, *Ty. angusticheles* Kudinova-Pasternak, 1989, and a third species from N Atlantic waters, that is described here for the first time. Current knowledge on ‘long-bodied’ typhlotanaids with rounded pereonites is summarised and a taxonomical key for their identification provided.

## Introduction

**Figure 1:**
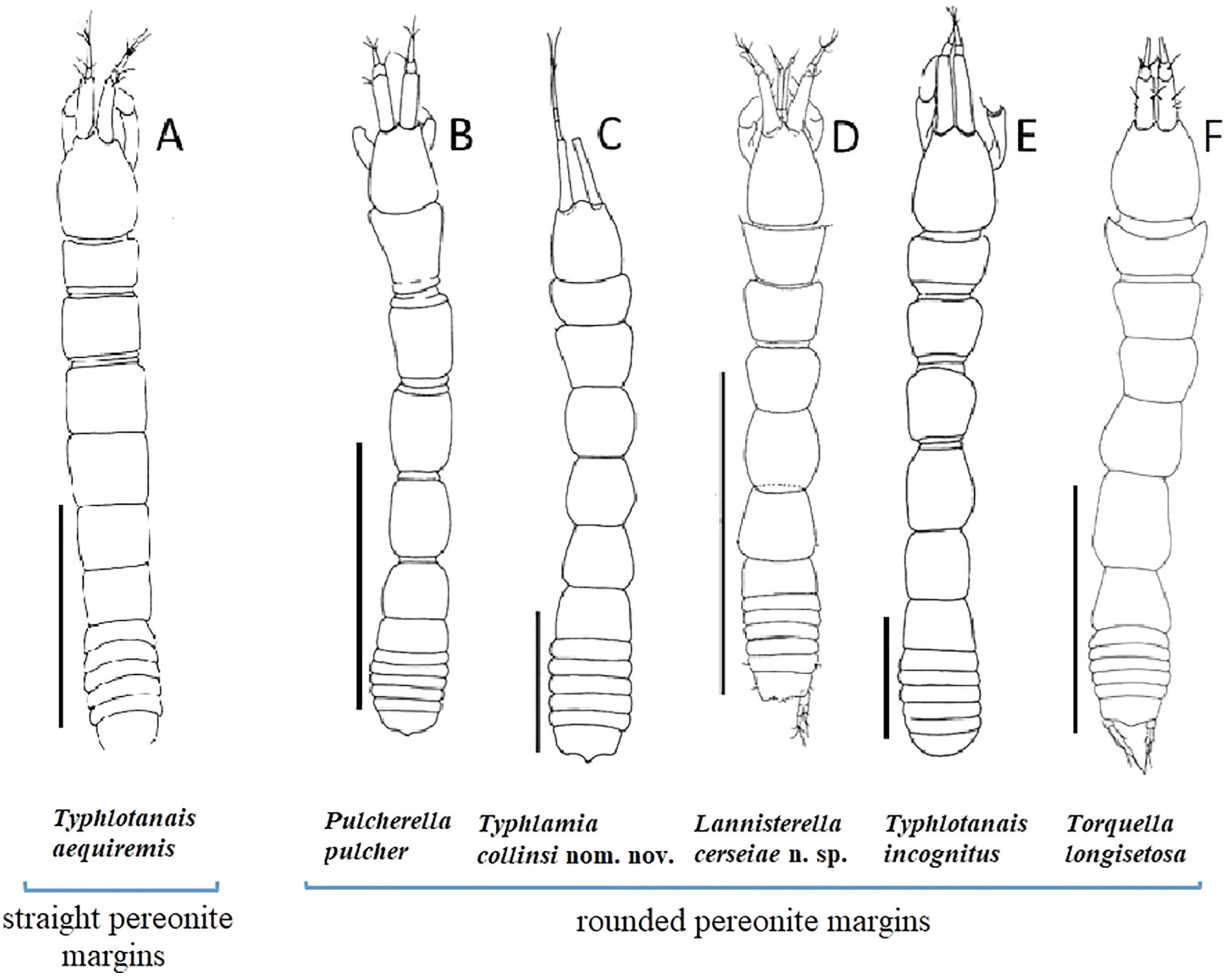
Comparison of body habitus. (A) *Typhlotanais aequiremis* (Lilljeborg, 1864). (B) *Pulcherella pulcher* (Hansen, 1913). (C) *Typhlamia collinsi* nom. nov. (D) *Lannisterella cerseiae* n. sp. (E) *‘variabilis’* group: *Typhlotanais incognitus* Larsen, Błażewicz-Paszkowycz & Cunha, 2006. (F) *Torquella longisetosa* (Kudinova-Pasternak, 1990) (from: Błażewicz-Paszkowycz, 2007a; Larsen, Błażewicz-Paszkowycz & Cunha, 2006). Scale: 1 mm.

*Typhlotanais* Sars, 1882 (Crustacea: Tanaidacea) is a diverse genus established by Sars (1882) about two decades after Lilljeborg (1864) discovered its first member and type species—*Tanais aequiremis* Lilljeborg, 1864—on north off Ireland (Lilljeborg, 1864; Sars, 1882). The definition of the genus comprises characters shared by many deep water tanaids such as three-article antennules and complete lack of eyes (Sieg, 1983, 1984). This short and elusive diagnosis is the main reason why *Typhlotanais* quickly became a hotchpotch taxon comprising a handful of diverse species (Sars, 1899; Hansen, 1913; Vanhöffen, 1914; Kudinova-Pasternak, 1966, 1984, 1990; Lang, 1968; Shiino, 1970). The excellent illustrations of Sars (1899) clearly show that *Typhlotanais aequiremis* (Lilljeborg, 1864) has a long body with distinctly straight pereonites (Fig. 1A). Morphological differences between the type species and the other putative *Typhlotanais* species were so apparent that, during the second half of the 20th century, four genera (*Meromonakantha* Sieg, 1986a, *Paratyphlotanais* Kudinova-Pasternak & Pasternak, 1978, *Peraeospinosus* Sieg, 1986a, and *Typhlotanoides* Sieg, 1983) and the family Typhlotanaidae Sieg, 1984 itself were erected (Kudinova-Pasternak & Pasternak, 1978; Sieg, 1983, 1984, 1986a). *Typhlotanais* currently includes 53 species (44% of 123 typhlotanaid species known) and it is the most speciose out of 16 typhlotanaid genera (Gellert, Palero & Błażewicz, 2022; WoRMS, 2022; Lubinevsky, Tom & Bird, 2022).

Kudinova-Pasternak (1985) reported four new Typhlotanaidae species from the RV *Vitjaz* expedition at the base of the Great-Meteor Seamount, North Atlantic, namely *Typhlotanais sandersi* Kudinova-Pasternak, 1985, *Paraleptognathia bacescui* Kudinova-Pasternak, 1985, *Paratanais hessleri* Kudinova-Pasternak, 1985, and *Pseudotanais siegi (P. diegi sic*!) Kudinova-Pasternak, 1985, and deposited type material for all those taxa at the Museum of Zoology in Moscow (Kudinova-Pasternak, 1985). According to the original description (in Russian), the holotype of *Ty. sandersi* was fully dissected and kept in slides (cat. No. Mh:4) before drawing its appendages. Those slides could not be found just two decades later though, so Błażewicz-Paszkowycz (2007a) decided to redescribe *Ty. sandersi* based on a second, undissected specimen, found in a jar labelled as “holotype cat. No. Mh:4” (Błażewicz-Paszkowycz, 2007a: p. 90–93, figs. 50–51). The specimen that Błażewicz-Paszkowycz found and illustrated was morphologically distinct from *Ty. aequiremis* (type species of *Typhlotanais*), so she decided to erect a new genus (*i.e*., *Typhlamia*) to accommodate what she called *Typhlamia sandersi* (Kudinova-Pasternak, 1985) along with *Tm. mucronata* (Hansen, 1913) and *Tm. bella* Błażewicz-Paszkowycz (2007a).

After a new species of *Typhlamia* Błażewicz-Paszkowycz, 2007a was collected from Northwest Pacific waters (Gellert, Palero & Błażewicz, 2022), detailed morphological analysis and literature revision has revealed that the original description of *Ty. sandersi* by Kudinova-Pasternak (1985) differed from the redescription made by Błażewicz-Paszkowycz (2007a). A thorough morphological analysis of ‘long-bodied’ typhlotanaids with rounded pereonite margins (*e.g*., *Typhlamia, Pulcherella, Torquella*), all different from *Ty. aequiremis*, has allowed us to conclude that the putative *Ty. sandersi* specimen studied by Błażewicz-Paszkowycz (2007a) was wrongly labelled and in fact represents a different species and genus from the *Ty. sandersi* described and figured by Kudinova-Pasternak (1985). This article aims to correct the taxonomic mixing of *Ty. sandersi* described by Kudinova-Pasternak (1985) and *Tm. sandersi sensu* Błażewicz-Paszkowycz (2007a) and to establish a new genus with the support of additional morphological and molecular evidence.

As a result of morphological analysis of long body typhlotanaids with rounded pereonite margins, a new morphological group ‘*variabilis*’ was distinguished, for which the definition is given here for the first time.

## Materials and Methods

### Literature data

Data on the morphology and morphometry of the ‘long-bodied’ typhlotanaids (body length ≥8.0 L:W) with rounded pereonites were collected from Bird (2004), Błażewicz-Paszkowycz (2007a), Błażewicz-Paszkowycz, Bamber & Jóźwiak (2013), Błażewicz-Paszkowycz et al. (2014), Gellert, Palero & Błażewicz (2022), Hansen (1913), Kudinova-Pasternak (1966, 1985, 1989, 1990), Kudinova-Pasternak & Pasternak (1978), Lang (1971), Larsen (2011), Larsen, Błażewicz-Paszkowycz & Cunha (2006), Larsen & Shimomura (2007), Sars (1882, 1899). The data gathered are shown in Table 1.

**Table 1:** Characteristic of ‘long-bodied’ typhlotanaids with rounded pereonites in dorsal view. Details of body habitus, cheliped, pereopod-1 and uropod are presented in Figs. 1, 2 and 4.

Genus/morpho-group		*Torquella* Błażewicz-Paszkowycz, 2007a	*Typhlamia* Błażewicz-Paszkowycz, 2007a	*Pulcherella* Błażewicz-Paszkowycz, 2007a	*Lannisterella* n. gen.	‘*variabilis*’ group
**Species included**		*Tq. angularis, Tq. eltaninae, Tq. galatheae, Tq. grandis, Tq. iberica, Tq. longisetosa, Tq. magdalensis, Tq. parangularis, Tq. rotundirostris*	*Tm. bella, Tm. genesis, Tm. mucronata, Tm. collinsi*	*P. juraszi, P. filatovae, P. pulcher, P. spiniventris*	*L. angusticheles, L. cerseiae, L. sandersi*	*Ty. incognitus, Ty. variabilis*
**Carapace**		Rounded (1.0–1.2 L:W)	Tapering proximally, short (1.0–1.2 L:W)	Tapering proximally, short (1.1 L:W)	Elongated (1.3 L:W)	Tapering proximally, short (1.2 L:W)
**Pereonite-1 proximal corners**		Proximally extended (‘collar’)	Proximally not extended	Proximally not extended	Proxilmally extended (‘collar’)	Proximally not extended
**Pereonite-1 lateral seta**		Present or absent	Absent	Absent	Present	Absent
**Antennule inner margin with**		Middle and distal setae	Two middle and one distal seta	middle and distal setae	Four long setae	Short middle and distal setae
**Antennule article-3**		Short	Long	Short	Short	Short
**Antennule with distal spur**		Present	Absent	Absent	Present	Absent
**Maxilliped**	**Basis**	Long	Short	Short	Long	Long
	**Basal seta**	Short	Long	Long	Short	Short
**Cheliped carpus ventral margin**		Rounded	Straight	Straight	Rounded	Straight
	**Minute (‘third’) seta**	Present	Absent	Absent	Present	Present
**Pereopod-1 carpus dorsodistal seta**		Short	Long	Long	Long/short	Short
**Pereopods 2–3 Propodus dorsodistal seta**		Long	Long	Long	Long	Short
	**Carpus (ornamentation)**	With short setae, spines, tubercles, rod setae	With setae and spine(s)	With setae only	With setae and spine(s)	With setae only
**Pereopods 4–6 carpus with clinging apparatus**		Surrounded by blunt spines	Not surrounded by blunt spines	Not surrounded by blunt spines	Surrounded by blunt spines	Not surrounded by blunt spines
**Pereopods 4–6 unguis**		Simple	Bifurcated	Bifurcated	Bifurcated	Bifurcated
**Uropod**	**Exopod (article number)**	Two-articled	One-articled	One-articled	Two-articled	Two-articled
	**Character**	Slender or short	Slender	Slender	Slender	Slender
	**Exopod/endopod**	Shorter	Shorter	Shorter	Shorter	Almost equal

### Genetic analyses

All sequences used in the molecular studies are from GenBank. Sequences from Gellert, Palero & Błażewicz (2022) were obtained from the University of Lodz Tanaidacea collection (GenBank accession numbers: ON310832-ON310845 for COI and ON255540-ON255555 for 18S rDNA; see Table 2). The molecular tree used in the current study come from publication by Gellert, Palero & Błażewicz (2022).

**Table 2:** Voucher codes for the museum specimens and GenBank accession numbers for the COI and 18S rDNA sequences used to build the molecular tree.

Species	Voucher	COI (GenBank accession numbers)	18S rDNA (GenBank accession numbers)	Reference
*Akanthophoreus* cf. *alba*	ITan165	–	ON255554	Gellert, Palero & Błażewicz (2022)
*Akanthophoreus* sp.	–	SRR14135881	ON255555	SRA database; Gellert, Palero & Błażewicz (2022)
*Paranarthrurella* sp.	ITan158	MK751352	MK804177	Błażewicz et al. (2019)
*Paranarthrurella* sp.	ITan160	MK751354	MK804178	
*Paranarthrurella* sp.	ITan162		MK804179	
*Paranarthrurella* sp.	ITan164	MK751357	–	
*Baratheonus roberti*	ZMHK-62910	ON310832	–	Gellert, Palero & Błażewicz (2022)
*Baratheonus roberti*	ZMHK-62914	ON310837	–	Gellert, Palero & Błażewicz (2022)
*Baratheonus roberti*	ZMHK-62912	ON310838	ON255543	Gellert, Palero & Błażewicz (2022)
*Baratheonus roberti*	ZMHK-62913	ON310841	ON255545	Gellert, Palero & Błażewicz (2022)
*Pulcherella pulcher*	ITan003	KJ934617	ON255546	Błażewicz-Paszkowycz et al. (2014), Gellert, Palero & Błażewicz (2022)
*Pulcherella pulcher*	ITan004	KJ934618	–	Błażewicz-Paszkowycz et al. (2014)
*Pulcherella pulcher*	ITan005	KJ934619	ON255547	Błażewicz-Paszkowycz et al. (2014), Gellert, Palero & Błażewicz (2022)
*Pulcherella pulcher*	ITan008	KJ934620	–	Błażewicz-Paszkowycz et al. (2014)
*Pulcherella pulcher*	ITan187	KJ934621	–	Błażewicz-Paszkowycz et al. (2014)
*Starkus sirene*	ZMHK-62865	ON310833	ON255540	Gellert, Palero & Błażewicz (2022)
*Starkus sirene*	ZMHK-62864	ON310834	ON255541	Gellert, Palero & Błażewicz (2022)
*Starkus sirene*	ZMHK-62844	ON310835	–	Gellert, Palero & Błażewicz (2022)
*Starkus sirene*	ZMHK-62847	ON310839	ON255544	Gellert, Palero & Błażewicz (2022)
*Torquella* cf. *grandis*	ITan062	KJ934614	–	Błażewicz-Paszkowycz et al. (2014)
*Torquella* cf. *grandis*	ITan091	KJ934613	–	Błażewicz-Paszkowycz et al. (2014)
*Typhlamia genesis*	ICUL7916	ON310836	ON255542	Gellert, Palero & Błażewicz (2022)
*Typhlamia genesis*	ZMHK-62928	ON310840	–	Gellert, Palero & Błażewicz (2022)
*Typhlamia genesis*	ZMHK-62919	ON310842	–	Gellert, Palero & Błażewicz (2022)
*Typhlotanais cornutus*	ITan041	MK751360	ON255551	Błażewicz et al. (2019), Gellert, Palero & Błażewicz (2022)
*Typhlotanais eximius*	ITan076	MK751361	–	Błażewicz et al. (2019)
*Typhlotanais finmarchicus*	WS 12845	–	MN337129	GenBank database
*Typhlotanais mixtus*	ITan011	ON310843	ON255548	Gellert, Palero & Błażewicz (2022)
*Typhlotanais mixtus*	ITan020		ON255549	Gellert, Palero & Błażewicz (2022)
*Typhlotanais mixtus*	ITan021	ON310844	ON255550	Gellert, Palero & Błażewicz (2022)
*Lannisterella cerseiae*	ITan050	KJ934599		Błażewicz-Paszkowycz et al. (2014)
*Lannisterella cerseiae*	ITan069	KJ934600	ON255552	Błażewicz-Paszkowycz et al. (2014), Gellert, Palero & Błażewicz (2022)
*Lannisterella cerseiae*	ITan099	KJ934601	ON255553	Błażewicz-Paszkowycz et al. (2014), Gellert, Palero & Błażewicz (2022)
*Typhlotanais variabilis*	ITan070	KJ934605	–	Błażewicz-Paszkowycz et al. (2014)
*Typhlotanais variabilis*	ITan077	ON310845	–	Gellert, Palero & Błażewicz (2022)

## Terminology and species description

Total body length (BL) was measured along the central axis of symmetry from the frontal margin to the end of pleotelson; body width (BW) was measured perpendicular to the main axis at the widest point of pereonite-3. Width and length of carapace, pereonites, pleonites, and pleotelson were measured on whole specimens. All measurements were taken using a digital camera connected to the microscope (Nikon Eclipse Ci-L) and the NIS-Elements View software (www.nikoninstruments.com).

The clinging apparatus is a system of various hooks, tubercles, thorns, and spines located on the carpus of pereopods 4−6 (Błażewicz-Paszkowycz, 2007a; Gellert et al., 2022). Unspecified setae in taxonomic descriptions are referred here as simple setae (= without ornamentation) by default. Besides, we recognize penicillate setae—with a distal tuft of setules and supracuticular articulation, and rod setae—distally inflated seta and with a terminal pore (Thomas, 1970; Garm, 2004). The short ventral seta situated besides two long setae on the cheliped carpus is called ‘third’ seta. The distal part of the cheliped basis, extending backwards, is referred here as ‘cheliped lobe’ (Larsen, 2005). The term ‘collar’ is used to refer the shape of pereonite-1, with a deeply concave anterior edge and lateral corners extended forwards (*e.g*., *Torquella* Błażewicz-Paszkowycz, 2007a). Błażewicz-Paszkowycz (2007a) proposed a classification of typhlotanaids into ‘short-bodied’ (body <6.0 L:W) and ‘long-bodied’ (body ≥8.0 L:W) taxa (see the Key for Typhlotanaidae genera and morpho-groups in Błażewicz-Paszkowycz, 2007a). The neuter is the post-manca stage, that cannot be classified as male or female. Two-letter genus abbreviations are used throughout the text to distinguish between genera: *Tq. = Torquella, Tm. = Typhlamia* and *Ty. = Typhlotanais*. Type material for the species described here is deposited in the Museum der Natur Hamburg, Germany (ZMHK) and ICUL/ITan numbers correspond to unique identifiers from the Invertebrates Collection held at the University of Lodz, Poland.

## Results and interpretation

The recent discovery of a new species of *Typhlamia* from the Kuril-Kamchatka Trench (Gellert, Palero & Błażewicz, 2022) and the re-evaluation of *Typhlotanais* sp. A from Błażewicz-Paszkowycz et al. (2014) uncovered significant genetic and morphological differences between *Typhlamia* and the morphogroup of species including the original holotype description and drawings of *Typhlotanais sandersi* Kudinova-Pasternak, 1985. Although types of all taxa described by Kudinova-Pasternak were deposited at the Zoological Museum in Moscow after her retirement (R.K. Kudinova-Pasternak in 2004, personal communication), all microscopic slides made for studying and illustrating morphological details were disposed of, even those with holotype appendages. For this reason, most of the type collection studied by Kudinova-Pasternak (except undissected type material) should be considered lost. This situation is particularly critical for *Ty. sandersi* Kudinova-Pasternak (1985), of which a single specimen was dissected after the habitus was illustrated.

According to Kudinova-Pasternak (1985), two *Typhlotanais* specimens were collected from the same station (st. 162) during the RV *Vitjaz* expedition at the base of the Great-Meteor Seamount: the holotype of *Ty. sandersi* described and drawn in the text and another individual identified as *Typhlotanais mucronatus* Hansen, 1913 (currently *Typhlamia mucronata* (Hansen, 1913)). The latter was not dissected by Kudinova-Pasternak because it was assigned to a known species and, most likely, it was kept intact at the Zoological Museum of Moscow State Lomonosov University within the original jar. Indeed, an intact specimen was later found by Błażewicz-Paszkowycz (2007a) during her revision of Typhlotanaidae taxa in a vial labelled “*Typhlotanais sandersi* n. sp., holotype, Mh 4”, and Błażewicz-Paszkowycz (2007a) wrongly considered it as type material of *Typhlotanais sandersi* Kudinova-Pasternak, 1985. In fact, the undissected specimen corresponded to the second *Typhlotanais* specimen from station 162, identified initially by Kudinova-Pasternak as *Typhlotanais mucronatus* Hansen, 1913.

**Figure 2:**
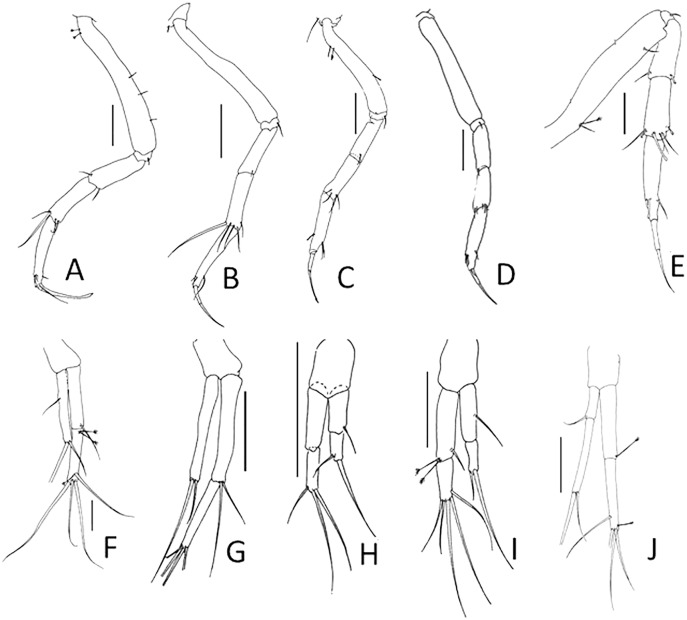
Comparison of pereopod-1 and uropod. (A, F) *Pulcherella pulcher* (Hansen, 1913). (B, G) *Typhlamia collinsi* nom. nov. (C, H) *Lannisterella cerseiae* n. sp. (D, I) *‘variabilis’* group: *Typhlotanais incognitus* Larsen, Błażewicz-Paszkowycz & Cunha, 2006. (E, J) *Torquella longisetosa* (Kudinova-Pasternak, 1990) (from: Błażewicz-Paszkowycz, 2007a; Larsen, Błażewicz-Paszkowycz & Cunha, 2006). Scale: 0.1 mm.

A careful comparison of their morphology, based on the original drawings, bring us to the conclusion that the true *Typhlotanais sandersi* (holotype) and the specimen referred by Błażewicz-Paszkowycz (2007a) as *Typhlamia sandersi* (Kudinova-Pasternak, 1985) represent two distinct taxa. All species of *Typhlamia* present an elongated third antennular article with particularly long distal setae (Fig. 1C), and the intact specimen is undoubtedly close to *Ty. mucronatus* Hansen, 1913; see Błażewicz-Paszkowycz, 2007a: 90–93). The absence of this antennular feature in the holotype of *Typhlotanais sandersi* illustrated by Kudinova-Pasternak (1985: page 54) drew our attention first, and further analysis revealed other significant differences in the uropods (exopod unarticulated and about half the endopod length in *Typhlamia* (Fig. 2G), whereas it is clearly biarticulated and subequal to endopod in the holotype) and cheliped carpus or pereonite-1 setation, among other features (see Table 1).

**Figure 3:**
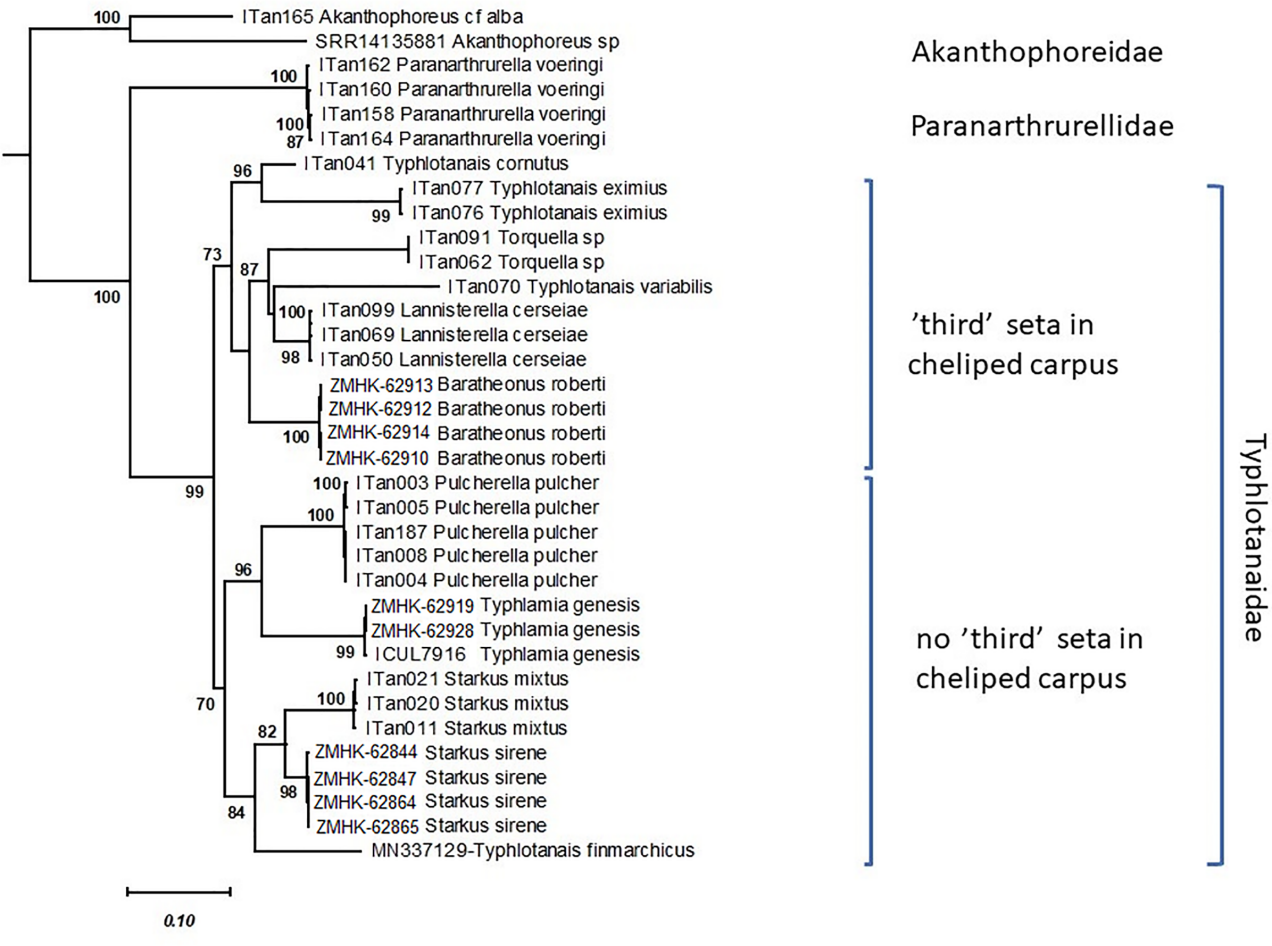
Maximum likelihood evolutionary tree of Typhlotanaidae species inferred from the COI and 18S concatenated alignment. Only statistically significant bootstrap values (>70%) are shown. Modified after Gellert, Palero & Błażewicz, 2022.

The morphological revision of ‘long-bodied’ typhlotanaids with rounded pereonites revealed that *Typhlotanais sandersi* Kudinova-Pasternak, 1985 is morphologically close to *Typhlotanais angusticheles* Kudinova-Pasternak, 1989 and *Typhlotanais* sp. A (Błażewicz-Paszkowycz et al., 2014) (Table 3). Those three species most likely belong to a new, undescribed, genus, because they are apparently different from both *Typhlamia* and *Typhlotanais aequiremis* Lilljeborg, 1864 (type species of *Typhlotanais*). The combination of features such as the long body and round pereonites distinguishes these three species from other Typhlotanaidae genera. Indeed, the molecular comparison of species of *Typhlamia genesis* and *Typhlotanais* sp. A (morphologically close to *Ty. sandersi*) confirmed that both taxa belong to genetically distinct clades (Fig. 3), giving further support for the establishment of a new genus, herein named *Lannisterella* n. gen.

**Table 3:** Distribution of *Typhlamia* species.

Species	Expedition	Area	Station	Latitude	Longitude	Depth [m]	Reference
*Tm. bella*	ANDEEEP-3 XXII	Weddell Sea	PS67/81-8-E	70°32.19′S	14°35.13′W	4,392–4,385	Błażewicz-Paszkowycz (2007a)
			PS 67/81-8-E	70°32.19′S	14°35.13′W	4,392–4,385	
	ANDEEEP-3 XXII	W off Antarctic Peninsula	PS 67/154-9	62°31.36′S	64°39.25′W	3,804–3,808	
*Tm. genesis*	KuramBio I	Kuril-Kamchatka Trench	1-10	43°57.92′N	157°23.76′E	5,423–5,429	Gellert, Palero & Błażewicz (2022)
			6-12	42°32.68′N	154°1.27′E	5,304–5,307	
			9-9	40°38.79′N	150°59.97′E	5,399–5,408	
			9-12	40°38.71′N	150°59.72′E	5,392–5,397	
			10-9	41°15.66′N	150°5.70′E	5,265–5,643	
			10-12	41°8.44′N	150°5.53′E	5,249–5,262	
			12-4	39°46.72′N	147°11.90′E	5,215–5,228	
	KuramBio II	Kuril-Kamchatka Trench	8	43°51.698′N	151°45.85′E	5,103–5,109	
			10	43°51.810′N	151°46.54′E	5,103–5,188	
			86	44°56.784′N	151°6.009′E	5,534–5,630	
			87	44°58.024′N	151°5.589′E	5,640–5,465	
	Vitjaz	Kuril-Kamchatka Trench	5617	45°32′N	153°46′E	6,740–6,710	Kudinova-Pasternak (1970)
			5624	45°22′N	154°00′E	5,200–5,240	
			5634	44°17′N	149°33′E	4,690–4,720	
		Gulf of Alaska	6109	56°14′N	139°44′W	3,460–3,450	Kudinova-Pasternak (1973)
*Tm. mucronata*	Ingolf	N Iceland	120	67°29′N	11°32′W	1,620	Hansen (1913)
			119	67°53′N	10°19′W	1,848	
		Jan Mayen	177	69°13′N	8°23′W	1,835	
	BIOGAS	Bay of Biscay	3	47°33.90′N	09°38.40′W	3,992–4,260	Holdich & Bird (1985)
*Tm. sandersi*	Vitjaz	North Atlantic	162	29°50′N	28°08′6E	3,080–3,140	Kudinova-Pasternak (1985)

**Figure 4:**
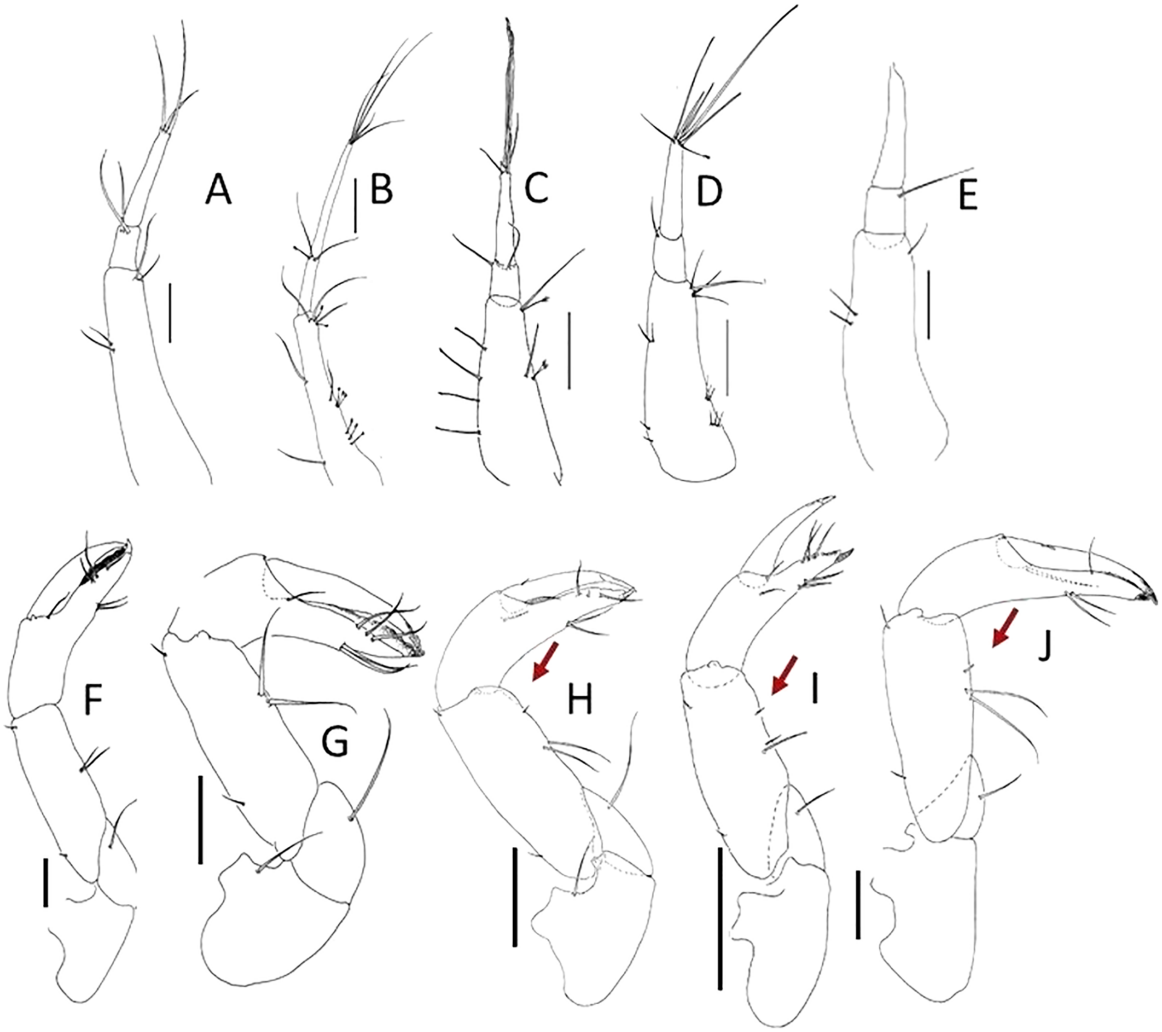
Comparison of antennule and cheliped. (A, F) *Pulcherella pulcher* (Hansen, 1913). (B, G) *Typhlamia collinsi* nom. nov. (C, H) *Lannisterella cerseiae* n. sp. (D, I) *‘variabilis’* group: *Typhlotanais incognitus* Larsen, Błażewicz-Paszkowycz & Cunha, 2006. (E, J) *Torquella longisetosa* (Kudinova-Pasternak, 1990) (from: Błażewicz-Paszkowycz, 2007a; Larsen, Błażewicz-Paszkowycz & Cunha, 2006). Scale: 0.1 mm.

Of the ‘long-bodied’ Typhlotanaidae with a developed clinging apparatus on pereopods 4–6 previously known, only a few taxa have rounded pereonite margins. These include the three genera—*Torquella, Pulcherella* and *Typhlamia*, and two *Typhlotanais* species which have very conservative mouth parts, but share several unique morphological characters—*Ty. variabilis* Hansen, 1913 and *Ty. incognitus* Larsen, Błażewicz-Paszkowycz & Cunha, 2006 (*‘variabilis’* morpho-group) (Hansen, 1913; Larsen, Błażewicz-Paszkowycz & Cunha, 2006). The morphological features that allow these genera and species-group to be distinguished from *Lannisterella* n. gen. are summarized in Table 1, and Figs. 1, 2 and 4.

## Taxonomy

**Genus** *Typhlamia* Błażewicz-Paszkowycz, 2007a

**Diagnosis** (Błażewicz-Paszkowycz, 2007a; amended): Body long, about 8.0–9.0 L:W. Carapace tapering proximally, short (1.0–1.2 L:W). Proximal corners not extended; lateral seta absent. Antennule inner margin with two medial and one distal setae; article-3 long; distal spur absent. Maxilliped basal seta long. Cheliped carpus ventral margin straight; ‘third’ seta absent. Pereopod-1 carpus dorsodistal with long seta; pereopods 2–3 propodus with long distodorsal seta, carpus with setae and spine(s); pereopods 4–6 carpus with two rounded/blunt cusps instead of prickly tubercles; pereopods 4–6 unguis bifurcated. Uropod slender; exopod one-articled.

**Type species:** *Typhlamia bella* Błażewicz-Paszkowycz, 2007a, by designation.

**Species included:** *Typhlamia bella* Błażewicz-Paszkowycz, 2007a; *Typhlamia genesis* Gellert, Palero & Błażewicz, 2022, *Typhlamia mucronata* (Hansen, 1913), *Typhlamia collinsi* Błażewicz nom. nov.

**Remarks:** Of all the ‘long-bodied’ of Typhlotanaidae with rounded pereonite margins, only *Typhlamia* and *Pulcherella* have slender uropods with one-articled exopod (Table 1, Figs. 2F, 2G). Furthermore, both genera have long dorsodistal seta on the pereopod-1 carpus. In contrast to *Pulcherella*, members of *Typhlamia* have long third article in the antennule provided with long distal setae (Figs. 4A, 4B).

***Typhlamia bella* Błażewicz-Paszkowycz, 2007a**

*Typhlamia bella*—Błażewicz-Paszkowycz (2007a): 6, 87, 90, 92, 94–98.

**Distribution:** Species only known from type locality: Weddell Sea, Antarctic (70°32.02′–70°32.19′S 14°35.05′–14°35.13′W) from 4,392–4,385 m depth, which is the deepest record of this species in the Atlantic. Another, much shallower record from 62°31.47′–62°31.36′S 64°39.45′–64°39.25′W at 3,804–3,808 m depth is doubtful (see Błażewicz-Paszkowycz, 2007a).

***Typhlamia mucronata* (Hansen, 1913)**

*Typhlotanais mucronatus*—Hansen (1913): 37, 131, 132, plate 4, 5; Nierstrasz (1913): 38; Stephensen (1932): 350; Shiino (1970): 275, 289; Holdich & Bird (1985): 445; Larsen, Błażewicz-Paszkowycz & Cunha (2006): 25; Kudinova-Pasternak (1985): 63; Kudinova-Pasternak (1990): 94.

*Typhlamia mucronata*—Błażewicz-Paszkowycz (2007a): 6, 27, 87–90.

not *Typhlotanais mucronatus*—Kudinova-Pasternak (1985): 52, 53.

**Remarks**: *Typhlamia mucronata* was redescribed by Błażewicz-Paszkowycz (2007a) based on Hansen’s collection from HMS *Ingolf* cruise (syntypes: CRU 3916, CRU 7423, CRU 3885) held in the Natural History Museum of Copenhagen.

**Distribution:** North of Iceland (67°29′N 11°32′W and 67°53′N 10°19′W) and Jan Mayen (69°13′N 8°23′W) from 1,618–1,847 m depth (Hansen, 1913; Lang, 1970); Bay of Biscay (47°33.90′N 09°38.40′W) from 3,992–4,260 m depth (Holdich & Bird, 1985), and Walvia Seamount (25°37′6″S 5°19′2″E) from 2,270 m depth (Kudinova-Pasternak, 1990).

***Typhlamia collinsi* Błażewicz nom. nov.**

LSID urn:lsid:zoobank.org:act:EC9D55F0-683F-4F0B-8487-ECEC22A33790

*Typhlotanais mucronatus*—Kudinova-Pasternak (1985): 52, 53.

*Typhlamia sandersi* (Kudinova-Pasternak, 1985)—Błażewicz-Paszkowycz (2007a): 6, 87, 90–93.

**Remarks:** *Typhlamia collinsi* description was based on a single specimen deposited at the Zoological Museum in Moscow (Błażewicz-Paszkowycz, 2007a) (see results section).

**Distribution:** Species known only from the type locality at Great-Meteor Seamount—North Atlantic (29°50′N 28°08′6E) at depths of 3,080–3,140 m (Kudinova-Pasternak, 1985).

***Typhlamia genesis* Gellert, Palero & Błażewicz (2022)**

*Typhlotanais mucronatus*—Kudinova-Pasternak (1970): 348, 379–380; Kudinova-Pasternak (1973): 153.

*Typhlamia mucronata*—Błażewicz-Paszkowycz (2007b): 102; Stępień, Pabis & Błażewicz (2019): 178: 3; Błażewicz et al. (2020): 474, 485–487.

*Typhlamia genesis*—Gellert, Palero & Błażewicz (2022).

**Distribution:** Species known from North Pacific waters. Reports from Kurile-Kamchatka Trench by Kudinova-Pasternak (1970) and Gellert, Palero & Błażewicz (2022) at depths from 4,690 to 6,740 m, and in much shallower waters, between 2,340–3,450 m, in the Gulf of Alaska. The depth range for *Typhlamia genesis* (as *Typhlotanais mucronatus*) given by Kudinova-Pasternak (1970) must be incorrect (4,840–6,675 m), but this mistake was repeated in latter papers (Błażewicz-Paszkowycz, 2007b; Stępień, Pabis & Błażewicz, 2019; Błażewicz et al., 2020) (Table 3).

**Genus *Lannisterella* n. gen.**

LSID urn:lsid:zoobank.org:pub:508FFE15-7F76-4125-9485-D7974A93BCD9

**Diagnosis:** Body long (7.0–8.0 L:W). Carapace elongated (1.3 L:W). Proximal corners extended (form ‘collar’); pereonite-1 lateral seta present. Antennule inner margin with four long setae*; article-3 short; distal spur present. Maxilliped basal seta short. Cheliped ventral margin rounded, with small ‘third’ seta. Pereopod-1 carpus short dorsodistal seta; pereopods 2–3 propodus distodorsal seta long; carpus with setae and spine(s); pereopods 4–6 carpus with clinging apparatus surrounded by blunt spines; pereopods 4–6 unguis bifurcated*. Uropod slender; exopod two-articled.

*Figures by Kudinova-Pasternak (1985) do not show setae on antennules, one of the carpal setae is longer than the others, unguis is simple.

**Etymology:** Named after the *Lannister* family, one of the Great Houses of Seven Kingdoms, from George R. R. Martin’s novel, *Game of Thrones*.

**Type species:** *Lannisterella cerseiae* n. sp.

**Gender:** feminine.

**Species included:** *Lannisterella angusticheles* (Kudinova-Pasternak, 1989); *Lannisterella sandersi* (Kudinova-Pasternak, 1985); *Lannisterella cerseiae* n. sp.

**Remarks:** The genus *Lannisterella* is a ‘long-bodied’ typhlotanaid with rounded pereonite margins and 2-article uropods rami (Figs. 1D and 2H). *Lannisterella* has rounded (not parallel) and smooth (not corrugated) pereonite margins like *Typhlamia*, *Torquella* and two species from the genus *Typhlotanais* (*‘variabilis’* group: *Typhlotanais variabilis* and *Typhlotanais incognitus*, see below). *Lannisterella* has uropodal exopod with two articles (one uropod exopod in *Typhlamia* and *Pulcherella*).

*Lannisterella, Torquella* and the *‘variabilis’* group have similar uropods. The *‘variabilis’* group is distinguished by clearly separated pereonites 1–3 (Fig. 1E), lacking the distal spur in antennule article-3 (Fig. 2D), short dorsodistal seta in pereopods 2–3 propodus and simple clinging apparatus on pereopods 4–6 (no blunt spines surrounding prickly tubercles).

Those characters allow to distinguish members of the *‘variabilis’* group from *Lannisterella* and *Torquella*, which pereonites 1–3 are not separated (Figs. 1D, 1F), antennule have distal spur (Figs. 4C, 4E), pereopods 2–3 propodus have a long dorsodistal seta and pereopods 4–6 carpal prickly tubercles are surrounded by blunt spines. *Lannisterella* can be distinguished from *Torquella* by the presence of four distinct setae on antennule article-1 (Fig. 4C), pereopods 1–3 lack rod setae, and pereopods 4–6 have simple unguis. In contrast, *Torquella* has few medial and distal setae on antennule article-1 (Fig. 4E), pereopods 1–3 articles bear distinct rod setae and pereopods 4–6 have bifurcated unguis.

***Lannisterella angusticheles* (Kudinova-Pasternak, 1989) n. comb.**

*Peraeospinosus angusticheles—*Kudinova-Pasternak (1989): 28, 30–33.

*Typhlotanais angusticheles—*Błażewicz-Paszkowycz (2007a): 5, 46; Błażewicz-Paszkowycz et al. (2014): 443.

**Diagnosis:** Antennule article-3 subequal to pereonite-2. Cheliped carpus short (2.1 L:W). Antennule article-3 long (10 L:W) (see Table 4). Pereopod-1 with three dorsodistal setae.

**Table 4:** Morphological features distinguishing *Lannisterella* species.

Species	*L. cerseiae*	*L. sandersi*	*L. angusticheles*
**Antennule article-3**	Short (7.0 L:W)	Short (6.2 L:W)	Long (10 L:W)
**Cheliped carpus**	Long (2.6 L:W)	Long (2.7 L:W)	Short (2.1 L:W)
**Pereonite-1 to pereonite-2**	Subequal	Shorter	Subequal
**Pereopod-1 propodus with dorsodistal seta**	One	Two	Three
**Pereopod-2 carpus ornamentation**	Two setae and one spine	Six setae and one spine	Data not available
**Uropod endopod**	6.0 L:W	8.0 L:W	Data not available

**Remarks:** *Lannisterella angusticheles* is distinguished from other members of the genus *Lannisterella* by having cheliped carpus 2.1 L:W (2.7 L:W in *L. sandersi* and 2.6 L:W in *L. cerseiae*). Pereopod-1 with three dorsodistal setae in *L. angusticheles*, two and one respectively in *L. sandersi* and *L. cerseiae*.

***Lannisterella sandersi* (Kudinova-Pasternak, 1985) n. comb.**

**Synonyms:** *Typhlotanais sandersi—*Kudinova-Pasternak (1985) 120: 52–55, Fig. 1.

**Diagnosis:** Antennule article-3 shorter than pereonite-2. Cheliped carpus long (2.7 L:W). Antennule article-3 short (6.2 L:W) (see Table 4). Pereopod-1 with two dorsodistal setae. Pereopod-2 carpus with six setae and one spine. Uropod endopod 8.0 L:W.

**Remarks:** *Lannisterella sandersi* is distinguished from its two congeners, by a relatively short (7.0 L:W) antennule article-3 (10 L:W in *L. angusticheles*), long (8.0 L:W) uropodal endopod (6.0 L:W in *L. cerseiae*) and presence of six setae in pereopod-2 carpus (two setae in *L. cerseiae*).

***Lannisterella cerseiae* n. sp.**

(Figs. 5 and 6)

**Figure 5:**
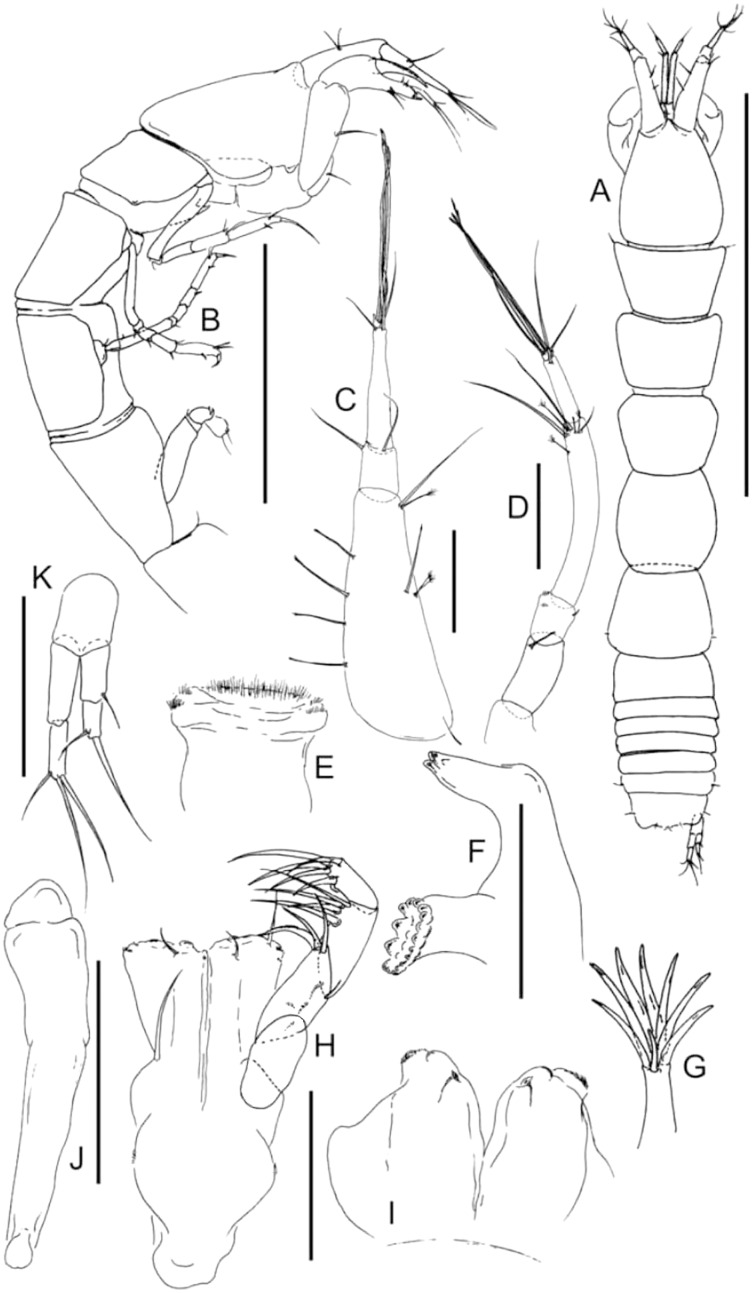
*Lannisterella cerseiae* n. sp., neuter. (A) Dorsal view. (B) Lateral view. (C) Antennule. (D) Antenna. (E) Labrum. (F) Right mandible. (G) Maxillule endite tip. (H) Maxilliped. (I) Labium. (J) Epignath. (K) Uropod. Scale: A–B = 1 mm, C–K = 0.1 mm (from Błażewicz-Paszkowycz et al., 2014).

**Figure 6:**
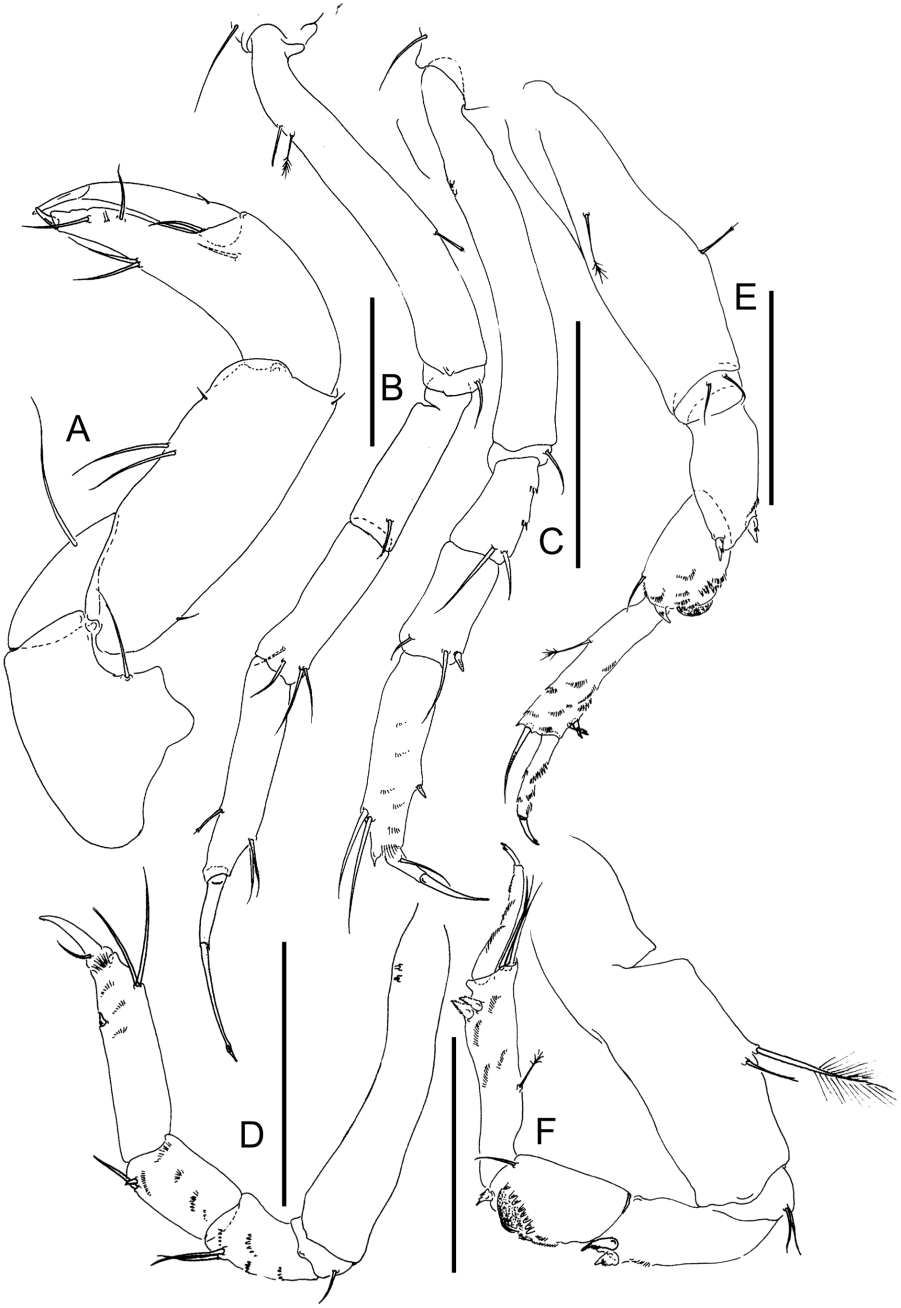
*Lannisterella cerseiae* n. sp., neuter. (A) Cheliped. (B) Pereopod-1. (C) Pereopod-2. (D) Pereopod-3. (E) Pereopod-4. (F) Pereopod-6. Scale: = 0.1 mm (from Błażewicz-Paszkowycz et al., 2014).

LSID urn:lsid:zoobank.org:pub:508FFE15-7F76-4125-9485-D7974A93BCD9

*Typhlotanais* sp. A: Błażewicz-Paszkowycz et al. (2014): 418, 421–423, 443–449.

**Studied material:**

Holotype, neuter (ZMH-K44196), IceAGE St. 1072, 63°00.46′–63°01.10′N 28°04.09′–28°03.15′W, epibenthic sledge, depth 1,569–1,594 m, 9 September 2011.

Paratype: neuter (ZMH-K44194), the locality as for holotype; mature (swimming) male (ZMH-K44190), the locality as for holotype.

**Diagnosis:** Antennule article-3 subequal pereonite-2. Cheliped carpus 2.6 L:W. Antennule article-3 short (7.0 L:W). Pereopod-1 with one dorsodistal seta. Pereopod-2 carpus with two setae and one spine. Uropod endopod 6.0 L:W.

**Etymology:** Named after Cersei Lannister, Queen of the Seven Kingdoms from George R.R. Martin’s novel, *Game of Thrones*.

**Description of neuter,** length 1.7 mm, paratype (ZMH-K44196). Body (Figs. 5A, 5B) slender, 7.7 L:W. Cephalothorax 1.2 L:W, 1.6× pereonite-1; eyes absent. Pereonites 1−6: 0.7, 0.8, 1.0, 1.0, 0.9 and 0.5 L:W, respectively. Pereonite-1 trapezoidal, subequal pereonite-2, with distinct seta on each side; pereonite-2 rectangular, 0.8× pereonite-3; pereonite-3 square, 0.9× pereonite-4; pereonite-4 square, 1.1× pereonite-5; pereonite-5 trapezoidal, 2.0× pereonite-6; pereonite-6 trapezoidal. Pleon 0.2× total BL; pleonites 1−5: all the same size—0.2 L:W. Pleotelson 2.8× pereonite-6.

Antennule (Fig. 5C) as long as cephalothorax; article-1, 0.7 of antennule length, 3.0 L:W, with four setae along the article, two penicillate setae and one medial long and penicillate and long setae distally; article-2, 1.4 L:W, 0.2× article-1, with two distal setae (inner and outer setae); article-3, 7.0 L:W, 2.3× article-2, with two short and five long setae distally.

Antenna (Fig. 5D) six-articled; article-1 fused with the body; article-2, 2.4 L:W, with distal seta; article-3, 1.4 L:W, 0.6× article-2, with short distal seta; article-4, 6.3 L:W, 3.6× article-3, with two short, two long (longer than article-5) and three penicillate setae distally; article-5, 5.0 L:W, 0.4× article-4, with long seta; article-6 1.0 L:W, with one short and four long distal setae.

Mouthparts. Labrum (Fig. 5E) hood-shaped, with rounded, setose distal margin. Right mandible (Fig. 5F) incisor with two lobes without *lacinia mobilis*. Labium (Fig. 5I) with distolateral corner finely setose. Maxillule (Fig. 5G) endite with eight terminal spines, one innermost spine shorter than the others. Epignath (Fig. 5J) simple distally. Maxilliped (Fig. 5H) basis with long seta, shorter than endites; endites unfussed, with two small gustatory cusps, seta on outer margin and seta on inner margin; palp with four articles: article-1 naked; article-2 with three inner setae and microtrichia on outer margin and outer seta; article-3 with four inner setae; article-4 with six inner distal and outer setae. Left mandible and maxilla lost during the dissection.

Cheliped (Fig. 6A) basis separated from pereonite-1, slender; 1.9 L:W, with long seta; merus subtriangular, with long ventral seta; carpus 2.7 L:W, with two long setae and short seta ventrally, two short setae on dorsal margin—dorsodistal rod seta and subdistal seta; chela 1.8× carpus, 4.8 L:W; palm 0.7× fixed finger, with two setae near dactylus insertion (inner and outer surface); fixed finger with two long ventral setae; cutting edge with three setae and three very weak, blunt cusps distally; dactylus with a short dorsoproximal seta.

Pereopod-1 (Fig. 6B) walking type, overall 22.5 L:W; coxa with seta; basis 7.8 L:W with three (subproximal, penicillate and rod medial) setae; ischium with ventral seta; merus 3.3 L:W with one ventrodistal seta; carpus 3.7 L:W; 0.8× merus, with four setae distally; propodus 3.0 L:W, 1.2× carpus, with one rod dorsodistal and two ventrodistal setae; dactylus 0.7× unguis; dactylus and unguis 0.6× propodus; unguis simple.

Pereopod-2 (Fig. 6C) walking type; overall 17.5 L:W; coxa with seta; basis naked; ischium with ventral seta; merus 1.9 L:W, with two ventrodistal setae and numerous calcified microtrichia; carpus 1.8 L:W, 0.9× merus, with dorsodistal seta and long seta and spine ventrodistally; propodus 4.2 L:W, 1.2× merus and carpus combined, with two dorsodistal setae and ventrodistal spine; dactylus 2.0× unguis; dactylus and unguis together 0.4× propodus; unguis simple.

Pereopod-3 (Fig. 6D) walking type, overall 15.8 L:W; coxa with seta; basis 6.0 L:W, naked; ischium with ventral seta; merus 1.9 L:W, with two long ventrodistal setae and numerous calcified microtrichia; carpus 1.8 L:W, 0.9× merus, with one spine and seta ventrodistally; propodus 4.2 L:W, 1.8× carpus, with ventrodistal spine, two dorsodistal setae, and numerous microtrichia; dactylus 2.0× unguis, with seta; dactylus and unguis together 0.4× propodus; unguis simple.

Pereopod-4 (Fig. 6E) clinging type, overall 7.8 L:W; coxa absent; basis slender, 4.2 L:W, with penicillate and rod medial seta; ischium with two ventral setae; merus 2.5 L:W, with two spines distally; carpus 1.8 L:W, 0.8× merus, with prickly tubercles moderate size, surrounded by spines and dorsal seta and rod seta distally; propodus 4.4 L:W, with numerous calcified microtrichia, two ventrodistal spines, and penicillate seta dorsally; dactylus 5.0× unguis, both combined 0.7× propodus; unguis simple.

Pereopod-5 as pereopod-4.

Pereopod-6 (Fig. 6F) as pereopod-4, but propodus with three dorsodistal setae.

Uropod (Fig. 5K) basal article 1.6 L:W; exopod two-articled, 5.2 L:W, 0.7 endopod, exopod proximal article 2.7 L:W, with distal seta; distal article 0.6× proximal, one long and one robust seta distally; endopod proximal article 3.0 L:W, naked; distal article 0.6 L:W, tipped by three setae distally.

Swimming male: The description supported with drawings of body habitus and appendages was presented in paper by Błażewicz-Paszkowycz et al. (2014): 443–449.

**Remarks:** *Lannisterella cerseiae* n. sp. has antennule article-1 3.6 L:W, what distinguishes it from *L. angusticheles* (2.9 L:W) and *L. sandersi* (5.1 L:W). Moreover, it has pereonite-1 subequal to pereonite-2, (clearly shorter in *L. angusticheles*) and has pereopod-1 propodus with only one dorsodistal seta (two setae in *L. sandersi* and three setae in *L. angusticheles*) (Table 4).

**Distribution:** Type locality off Iceland (63°00.46′–63°01.10′N 28°04.09′–28°03.15′W) at 1,569–1,594 m depth.

## Identification key for neuters of *Lannisterella*

1. Antennule article-1 long (10 L:W). . . . . . . *L. angusticheles* (Kudinova-Pasternak, 1989)
Indian Ocean 3600–5047 m
- Antennule article-1 short (<6.0 L:W). . . . . . . . . . . . . . . . . . . . . . . . . . . . . . . . . . . . . . . . . . . . . . . . . . 2
2. Uropod endopod short (6.0 L:W). . . . . . . . . . . . . . . . . . . . . . . . . . . . . . . . . . . . . . . . . . . . . . . . . . *L. cerseiae* n. sp.
Iceland (N Atlantic) 1569–1594 m
- Uropod endopod long (8.0 L:W). . . . . . . . . . . . . . . *L. sandersi* (Kudinova-Pasternak, 1985)
Great Meteor Seamount (N Atlantic) 3080–3140 m

***Typhlotanais ‘variabilis’* group**

(Figs. 1E, 2D, 2I, 4D and 4I)

**Diagnosis:** Carapace narrowing proximally, short (1.2 L:W). Proximal corners not extended (not form ‘collar’); pereonite-1 lateral seta absent. Antennule inner margin with short medial and distal setae; article-3 short; distal spur absent. Maxilliped basal seta short. Cheliped ventral margin straight; ‘third’ seta present. Pereopod-1 carpus short dorsodistal seta; pereopods 2–3 propodus distodorsal seta short; carpus (ornamentation) with setae only; pereopods 4–6 carpus with clinging apparatus not surrounded by blunt spines; pereopods 4–6 unguis bifurcated. Uropod slender; exopod two-articled.

**Species included:** *Typhlotanais variabilis* Hansen, 1913; *Typhlotanais incognitus* Larsen, Błażewicz-Paszkowycz & Cunha, 2006.

**Remarks:** The *‘variabilis’* group is proposed for ‘long-bodied’ of Typhlotanaidae with prickly tubercles and rounded pereonite lateral margins (Table 1). At first glance, the *‘variabilis’* group may resemble *Lannisterella*, which also has slender cheliped carpus with ‘third’ seta and biarticulated uropod rami, and pereonites 1–3 clearly separated by flexible articulations. By this characters, antennule without distal spur, pereonite-1 without lateral seta and pereopods 4–6 carpus with prickly tubercles not surrounded by blunt spines, the members of the morpho-group are distinguished from *Lannisterella*, which have antennule with distal spur, pereonite-1 with lateral seta and pereopods 4–6 carpus with prickly tubercles surrounded by blunt spines. The first article of the antennule is also less setose, being supplied with fine medial and distal setae, where *Lannisterella* has four long setae at this article margin.

Currently two species are included within the *‘variabilis’* morpho-group. Both species can be distinguished by length of the dorsodistal seta in pereopods 4–5 propodus, which is long in *Ty. variabilis* (reaches tip of unguis) and short (reaches half of the dactylus) in *Ty. incognitus*.

Currently *Typhlotanais sensu* stricto includes only the species of the genus (*Ty. aequiremis*). The *‘variabilis’* group can be included in *Typhlotanais sensu* lato. It is recognised that continued research may in the future provide the basis for the establishment of a new genus

## Identification key for neuters of ‘long-bodied’ typhlotanaids with rounded pereonites

1. Uropod exopod two-articled. . . . . . . . . . . . . . . . . . . . . . . . . . . . . . . . . . . . . . . . . . . . . . . . . . . . . . . . . . 3
- Uropod exopod one-articled. . . . . . . . . . . . . . . . . . . . . . . . . . . . . . . . . . . . . . . . . . . . . . . . . . . . . . . . . . 2
2. Antennule twice long as carapace and long terminal setae. . . . . . . . . . . . . . . . . *Typhlamia*

- Antennule as long as carapace and short terminal setae. . . . . . . . . . . . . . . . . . . . . *Pulcherella*

3. Pereopods 2–3 propodus with short dorsodistal seta. . . . . . . . . . . . . . . . . *‘variabilis’* group
- Pereopods 2–3 propodus with long dorsodistal seta. . . . . . . . . . . . . . . . . . . . . . . . . . . . . . . . . . 4
4. Pereopods 1–3 with rod setae and pereopods 4–6 unguis simple. . . . . . . . . . . . *Torquella*

- Pereopods 1––3 without rod setae and pereopods 4––6 unguis bifurcated. . . . . . . . . . . . *Lannisterella* n. gen.

## Genetic data

The Maximum Likelihood tree shows that the typhlotanaid taxa analyzed can be split into two main groups based on the presence or absence of a ‘third’ seta on cheliped carpus (Fig. 3). This small seta is ontogenetically independent (present in all development stages) and observed even in old historical collections. The group without a third seta includes four genera with two subclades. The first subclade comprising *Pulcherella* and *Typhlamia*, two genera with rounded pereonite margins, long uropods with unarticulated exopod, slender cheliped, and bifurcated unguis in pereopods 4–6 (see Figs. 1, 2 and 4); and the second subclade including taxa with straight pereonite margins, *e.g*., *Starkus mixtus* (Hansen, 1913), *Starkus sirene* Gellert, Palero & Błażewicz, 2022 and *Typhlotanais finmarchicus* Sars, 1882. The group with a ‘third’ seta on the cheliped carpus includes species with ‘short-bodied’ (*Typhlotanais cornutus* and *Typhlotanais eximius*) and ‘long-bodied’, which are further split into taxa with rounded pereonite margins, two-articled uropod rami and collar-shape of pereonite-1 (*Lannisterella, Torquella* and *‘variabilis’* group), and *Baratheonus roberti* Gellert, Palero & Błażewicz, 2022 characterized by straight pereonite margins, simple pereonite-1 and unarticulated uropod rami.

## Discussion

The paradigm that the deep-sea ecosystem is continuous (no topographic barriers) and stable over time resulted in the widespread acceptance of large geographic ranges for deep sea taxa (Kudinova-Pasternak, 1970, 1973; Sieg, 1986b). Nevertheless, a new concept of the deep sea as a highly diverse ecosystem has emerged (Zardus et al., 2006; Etter & Bower, 2015), acknowledging that restricted gene flow and population connectivity might redefine the general idea of widely distributed deep-sea species, particularly among Peracarida (Brandt et al., 2012; Hilário et al., 2015; Jakiel, Stępień & Błażewicz, 2018; Jakiel, Palero & Błażewicz, 2019, 2020). The consequences of insufficient knowledge about typhlotanaid taxonomy or inaccurate research methods only become obvious after state-of-the-art methods (*e.g*., application molecular approach or powerful microscopy) are improved and the data is re-analyzed. Our revision of ‘long-bodied’ typhlotanaids with rounded pereonites showed Kudinova-Pasternak (1985) was not able to notice the fine morphological features that differentiate *Typhlamia mucronata* (Hansen, 1913) (*Typhlotanais mucronatus* in Kudinova-Pasternak, 1985) from earlier studied *Typhlamia genesis* (see synonyms this article) despite both taxa occupy different oceanic basins. Historical collections, which include type material or hold rare specimens from pioneering scientific expeditions exploring the ocean floor (Beddard, 1886; Hansen, 1913) are kept in museums and treated as most valuable objects (Frutos et al., 2022). Because of their rarity, newly discovered deep-sea species are not always described at once, but they are generally drawn or photographed and described without providing a name, waiting for more material to be compared and supporting the erection of a new taxon (Błażewicz-Paszkowycz & Larsen, 2005; Kavanagh, Frutos & Sorbe, 2015; Stępień et al., 2022). Such is the case of *Typhlotanais* sp. A from Błażewicz-Paszkowycz et al. (2014), which was illustrated, diagnosed, and kept unnamed until more material or new evidence could support the erection of a new species. Morphological revision of ‘long-bodied’ typhlotanaids with rounded pereonites, combined with newly obtained molecular data, allowed us to describe it here as *Lannisterella cerseiae* n. gen., n. sp.

New, overlooked, or mislabelled taxa can be discovered while working with historical collections. Some species are described only provisionally, waiting for a subsequent revision or the discovery of a sibling species (Corbari, Frutos & Sorbe, 2019; Segadilha, Serejo & Błażewicz, 2019). During revision of high-level taxa and examination of historical collections, re-examination and checking of original labels allow to amend specimen misidentifications and/or to correct the geographical coordinates (Frutos & Sorbe, 2010).

Correct taxonomic identification is a baseline for biological research and the analyses at each level of biological organization (*e.g*., organism, population, community, ecosystem). Its accuracy determines quality of further analyses, *e.g*., phylogenetic, biogeographic or ecological, therefore, failure in taxonomic identification may turn in erroneous results and lead to wrong conclusions of next-level analyses (Frutos et al., 2022; Kürzel et al., 2022). An integrative approach combining genetic and morphological data allowed us to reconstruct historical (literature) data for material that has been lost or erroneously reported. Its interpretation in the light of new and improved knowledge also allowed us to propose changes in Typhlotanaidae systematics and clarify several taxonomic uncertainties. A phylogenetic analysis based on morphological traits was attempted. Unfortunately, due to high plasticity and divergence, it was not possible to obtain a tree with acceptable parameters (*e.g*., Consistency Index, Bremer Support). The molecular analysis of Typhlotanaidae taxa presented here must be taken as a preliminary result that requires further studies, including larger number of genes and taxa. While accelerated climate change and plastics are affecting even the deepest parts of the ocean, an accurate estimation and understanding of the biodiversity in this virtually unknown environment is needed before irreversible changes happen.

## Supplemental Information

10.7717/peerj.14272/supp-1 Supplemental Information 1 18S sequences.

10.7717/peerj.14272/supp-2 Supplemental Information 2 CO1 sequences.

## Additional Information and Declarations

### DNA Deposition

The following information was supplied regarding the deposition of DNA sequences: The sequences are accessible at GenBank: ON310832-ON310845 for COI and ON255540-ON255555 for 18S rDNA.

### Data Availability

The following information was supplied regarding data availability: Three new individuals of *Lannisterella cerseiae* n. sp. are described in the article. The material for the species is deposited in the Museum der Natur Hamburg, Germany (ZMHK): - Holotype, neuter: catalogue number ZMH-K44196, COI (GenBank accession numbers) KJ934601, 18S rDNA (GenBank accession numbers) ON255553; - paratype, neuter: catalogue number ZMH-K44194, COI (GenBank accession numbers) KJ934600, 18S rDNA (GenBank accession numbers) ON255552; - mature (swimming) male: catalogue number ZMH-K44190, COI (GenBank accession numbers) KJ934599.

### New Species Registration

The following information was supplied regarding the registration of a newly described species: Publication LSID urn:lsid:zoobank.org:pub:508FFE15-7F76-4125-9485-D7974A93BCD9 *Lannisterella* genus LSID: urn:lsid:zoobank.org:pub:508FFE15-7F76-4125-9485-D7974A93BCD9 *Lannisterella cerseiae* LSID: urn:lsid:zoobank.org:act:A4B6718E-8D62-4B9C-B9B8-E8054155070D *Typhlamia collinsi* LSID: urn:lsid:zoobank.org:act:EC9D55F0-683F-4F0B-8487-ECEC22A33790.
